# Open-Access Mega-Journals: A Bibliometric Profile

**DOI:** 10.1371/journal.pone.0165359

**Published:** 2016-11-18

**Authors:** Simon Wakeling, Peter Willett, Claire Creaser, Jenny Fry, Stephen Pinfield, Valérie Spezi

**Affiliations:** 1 Information School, University of Sheffield, Sheffield, United Kingdom; 2 LISU, Loughborough University, Loughborough, United Kingdom; 3 School of the Arts, English and Drama, Loughborough University, Loughborough, United Kingdom; Universidad de las Palmas de Gran Canaria, SPAIN

## Abstract

In this paper we present the first comprehensive bibliometric analysis of eleven open-access mega-journals (OAMJs). OAMJs are a relatively recent phenomenon, and have been characterised as having four key characteristics: large size; broad disciplinary scope; a Gold-OA business model; and a peer-review policy that seeks to determine only the scientific soundness of the research rather than evaluate the novelty or significance of the work. Our investigation focuses on four key modes of analysis: journal outputs (the number of articles published and changes in output over time); OAMJ author characteristics (nationalities and institutional affiliations); subject areas (the disciplinary scope of OAMJs, and variations in sub-disciplinary output); and citation profiles (the citation distributions of each OAMJ, and the impact of citing journals). We found that while the total output of the eleven mega-journals grew by 14.9% between 2014 and 2015, this growth is largely attributable to the increased output of *Scientific Reports* and *Medicine*. We also found substantial variation in the geographical distribution of authors. Several journals have a relatively high proportion of Chinese authors, and we suggest this may be linked to these journals’ high Journal Impact Factors (JIFs). The mega-journals were also found to vary in subject scope, with several journals publishing disproportionately high numbers of articles in certain sub-disciplines. Our citation analsysis offers support for Björk & Catani’s suggestion that OAMJs’s citation distributions can be similar to those of traditional journals, while noting considerable variation in citation rates across the eleven titles. We conclude that while the OAMJ term is useful as a means of grouping journals which share a set of key characteristics, there is no such thing as a “typical” mega-journal, and we suggest several areas for additional research that might help us better understand the current and future role of OAMJs in scholarly communication.

## Introduction

Following the advent in 2006 of *PLOS ONE*, the very first open-access mega-journal (OAMJ), the last few years have seen the arrival of many others, such as *AIP Advances*, the *Open Library of the Humanities*, *SAGE Open* and *Scientific Reports*. An OAMJ is an open-access, online-only, peer-reviewed publication that is normally funded through pre-publication article processing charges (APCs). This is, of course, no different from many other open-access (OA) journals, but OAMJs have two further characteristics that differentiate them from conventional OA publications. Firstly, they cover very broadly defined subject domains, as against the highly specific focus that characterises a typical academic journal, whether OA or non-OA. Related to this is the scale of their operations, with some (but by no means all) OAMJs publishing far greater numbers of articles each year than would appear in a conventional journal. Secondly, they adopt a different approach to peer review, eschewing traditional approaches to selection on grounds of novelty and/or significance and instead publishing all submissions that the reviewers agree are technically sound. In this respect, an OAMJ can be regarded as a distribution mechanism rather than having the gatekeeper role associated with top-tier journals.

The radical nature of these differences has the potential to bring about substantial changes in the journal publishing industry and in scholarly communication more generally, and there is already a small but growing body of literature associated with various aspects of the OAMJ phenomenon [1]. In this article, we present a quantitative analysis of eleven OAMJs using established bibliometric methods that have been developed to describe the publication and citation profiles of conventional journals. These methods are discussed by Anyi *et al*. [2], who note that a bibliometric journal study provides a quantitative portrait of that journal in terms of characteristics such as growth of the journal over its lifetime, the geographic distribution of contributions to it, the most prolific authors and institutions, the most heavily cited articles (and why these articles had attracted so much interest) and the extent to which the journal is cited by journals outside its own particular subject domain *inter alia*. Journal analyses have been reported across a very wide range of disciplines: the reviews of Tiew [3] and of Anyi *et al*. [2] summarised nearly 200 such studies that had been published in the period 1969 to 2008.

The newness of OAMJs has meant that quantitative studies of them are far less common than of conventional journals, and we have been able to identify only four such publications to date. The first of these, by Fein [4], appeared in 2013 and was based on the analysis of a set of 28,252 documents (comprising not just articles, but also reviews and editorial matter etc.) that had been published in *PLOS ONE* in the period 2007–2011. This set was evaluated in terms of five different types of criterion, *viz* journal output, journal content, journal perception, journal citations, and journal management. Each of these criteria was considered using one or more metrics, with results presented for the numbers of articles per month, authors’ countries, tag clouds based on the words comprising article titles, the citing article’s author’s country and document type, citation rates broken down by document type and year, the time from submission to publication, and the composition of the editorial board. Burns [5] studied a small sample of 49 articles published in the first few months after the launch of *PeerJ* in 2012, focusing on the journal’s peer review procedures, author demographics, usage data (as estimated by altmetrics such as article downloads and social media references) and citations to the articles (where the wide range of journals citing the sample articles suggested that the latter are highly varied in the subjects that they discuss). The study by Solomon [6] involved a Web-based survey of 2,128 authors who had published in *BMJ Open*, *PeerJ*, *PLOS ONE* or *Sage Open*. The survey focused on the characteristics of mega-journal authors and of the papers they had submitted for publication, the reasons for their choice of journal, and their approach to the payment of APCs. Finally, Björk and Catani [7] compared the distributions of citations to articles published in *PLOS ONE* and *Scientific Reports* with the corresponding distributions for articles from several conventional journals (where the review process requires consideration of novelty and significance when deciding which articles should be accepted for publication). Little difference was observed in the two sets of distributions, leading the authors to wonder whether “simple, soundness-only” refereeing might be more widely adopted.

This paper seeks to establish the bibliometric profiles of eleven leading OAMJs as of early 2016. It is the first such overview of the field and thus establishes a baseline for future studies as these, and other, OAMJs evolve over the coming years. The purpose of the paper is to explore systematically the following four characteristics of mega-journals, all of which are commonly used in bibliometric analyses [2]:

1. Journal outputs: the number of articles published and changes in output over time
2. Author characteristics: author nationalities and institutional affiliations
3. Subject areas: the disciplinary scope of OAMJs, and variations in sub-disciplinary output
4. Citation profiles: the citation distributions of each OAMJ, and the impact of citing journals.

The next section of the paper describes the criteria that we have adopted to select OAMJs for analysis, and the data collection processes. We then present results for a single mega-journal–*BMJ Open*–primarily as a means of introducing the modes of analysis employed in this study. In the following parts we structure our analysis of mega-journals in four sections, each relating to an area of investigation–output, author characteristics, subject areas, and citation analysis.

## Methods

### OAMJ Selection Criteria

We have summarised the characteristics of OAMJs in the previous section but it is less easy to provide a precise definition, since different authors describe them in different ways [8–11], and some that have been described as mega-journals have occasioned concerns as to the nature of the peer review processes that were used [12,13]. The criteria put forward by Björk [10] provide arguably the most comprehensive way of describing an OAMJ, and we have used a modified form of these in the selection of the journals that are discussed in the main body of this paper. Björk presents two sets of criteria to characterise OAMJs. The four primary criteria are as follows: big publishing volume (or at least aiming to achieve this); peer review that takes account of scientific soundness only, without consideration of originality or significance; a broad subject area; and full open access with publication funded by APCs. Björk suggests that a journal must satisfy all of these primary criteria if it is to be regarded as an OAMJ, and it should additionally satisfy at least some of seven secondary criteria: a moderate level of APCs (for which a figure of $1500 or less is suggested); a reputable, high-prestige academic publisher; editorial control by academic, rather than publisher, editors; use of Creative Commons licenses so that materials (graphics, data etc.) can be re-used by readers without the need to obtain formal permission from the author(s); the use of altmetrics to provide post-publication evaluation of significance; provision of facilities to enable readers to input comments on the article; the inclusion of reviews–sometimes referred to as “portable reviews”—from other journals that had rejected the article prior to its publication in the OAMJ; and rapid publication (for which a figure of less than six months from submission is suggested).

An initial list of 63 potential OAMJs for the present study was obtained from literature sources, personal knowledge, and publisher information. Of these, 20 were identified that satisfied all of the four primary criteria listed above. However, in the context of the bibliometric analyses to be conducted here, there is a further, essential, criterion: the OAMJ must be indexed by *Web of Science* and/or *Scopus*, the two principal sources of curated publication and citation data. Furthermore, in order to be able to conduct meaningful citation analysis, we required these data to be available from 2013 or earlier (thereby excluding mega-journals launched in 2014 or later). Of the two databases, *Scopus* was found to provide better coverage, with data available for 11 of the 20 OAMJs, *viz AIP Advances*, *BMC Research Notes*, *BMJ Open*, *F1000*, *FEBS Open Bio*, *Medicine*, *PeerJ*, *PLOS ONE*, *SAGE Open*, *Scientific Reports* and *SpringerPlus*. The principal characteristics of these journals are detailed in Table 1 (with much greater detail on them relating to all of the Björk criteria being provided as Supporting Information S1 Appendix). Of these, three journals perhaps merit special mention. Although founded in 1922, the journal *Medicine* was until mid-2014 a traditional, highly selective subscription journal publishing between 30 and 50 articles per year. It then transitioned to a mega-journal model, with a “soundness only” review policy and a gold open-access economic model. It therefore offers a unique opportunity to evaluate how the shift to a mega-journal model has affected its bibliometric profile. *F1000 Research* operates a post-publication peer-review model, whereby all submissions that pass initial in-house checks are published, with formal peer-reviews added later by members of the F1000 community. Only articles that have received two “Approved” or one “Approved” plus two “Approved with Reservations” reviews are submitted for indexing in databases such as *PubMed*, *Web of Science* and *Scopus*. While some commentators suggest this model aids the publication of poor quality science [14], only articles indexed in *Scopus*, and therefore that have received two positive peer-reviews, are included in our analysis. Finally *SpringerPlus* is included in our analysis, despite Springer announcing in June 2016 that the journal was to close, with no new submissions being accepted. It should also be noted that while the eleven journals mentioned above are the focus of this paper, data relating to a number of non-mega-journals are included in the analyses for comparison purposes.

**Table 1:** OAMJs considered in this article.

Title	Publisher (website)	Subject area	Start	2014 JIF	2014 SNIP	Articles^(1)^
*AIP Advances*	American Institute of Physics	Physical sciences	2011	1.524	0.696	2,456
*BMC Research Notes*	Springer	Biology and medicine	2008	n/a	0.683	4,419
*BMJ Open*	British Medical Journal	Medicine	2011	2.271	1.043	3,807
*F1000 Research*	Faculty of 1000	Science, medicine and technology	2012	n/a	0.189	628
*FEBS Open Bio*	Elsevier (on behalf of the Federation of European Biochemical Societies)	Molecular and cellular life sciences	2011	1.515	0.516	365
*Medicine*	Wolters Kluwer	Medicine	1922 / 2014	5.723	3.149	1,983^(2)^
*PeerJ*	PeerJ	Biological and medical sciences	2013	2.112	0.818	1,480
*PLOS ONE*	Public Library of Science	Science and medicine	2006	3.234	1.034	141,614
*SAGE Open*	Sage	Social sciences and humanities	2011	n/a	0.174	922
*Scientific Reports*	Nature Publishing Group	Natural and clinical sciences	2011	5.578	1.402	18,056
*SpringerPlus*^(3)^	Springer	Science	2012	n/a	0.511	2,356

^**(1)**^ The total number of articles indexed in the *Scopus* database as either “Articles” or “Articles in Press” up to the end of 2015.

^**(2)**^ Since *Scopus* indexes articles both before and after *Medicine*’s transition to the mega-journal model, the total number of articles found in *Scopus* is 4,409. The figure included in the table refers only to articles published after the OAMJ transition.

^**(3)**^ Springer announced on 13^th^ June 2016 that they would no longer be accepting new submissions to the journal.

### Data Collection and Analysis

With the exception of some figures relating to subject area coverage, which were obtained directly from the relevant OAMJ websites, all data for this paper were obtained through Scopus.com. As previously discussed, *Scopus* was found to have slightly greater coverage of OAMJ titles than *Web of Science*. While research has shown that *Scopus* covers a higher proportion of journals in general than *Web of Science*, it is important to note the variations in coverage for different subject areas [15]. In particular, the coverage of social science and arts and humanities journals has been found to be significantly lower than for titles in the natural sciences and biomedical fields. Four forms of *Scopus* data were collected for the study in March 2016: the bibliographic records for articles published in the eleven mega-journals; aggregations of institutional affiliations and associated nationalities for each OAMJ, available through the “Analyze search results” function of *Scopus*; “Citation overviews” for each OAMJ, which includes the number of citations by year for each article published in the journal; and aggregations of Journal Name and Subject Area for citing articles, again available through the “Analyze search results” function. A limitation of *Scopus* is that a number of functions, including the downloading of citation data, are only available up to a maximum of 20,000 lines of data. A series of filters were therefore used to segment the outputs for high volume journals (e.g. *PLOS ONE* and *Scientific Reports*), and the resulting downloads subsequently merged. One important point to note is that *Scopus* data relating to author nationality is based on the location of the author’s affiliated institution. Thus results and discussion relating to “author nationality” in this paper should be understood in this context.

Initial descriptive statistics were calculated in MS Excel^®^, with subsequent statistical analysis conducted using IBM SPSS Statistics v22^®^. Formal statistical tests (the independent-samples t-test, Welch analysis of variance (ANOVA), and the post-hoc Games-Howell test) were used when appropriate. Graphical representations of cumulative citation distributions were created in MS Excel^®^, and show curves smoothed with standard Excel smoothing (based on a Catmull-Rom spline).

## Case Study of *BMJ Open*

We begin by presenting an analysis of one prominent mega-journal–*BMJ Open*. This is intended primarily to introduce the methods employed in our comparison of the eleven selected mega-journals, while also offering a profile of a journal that by two basic measures—article output and age as listed in Table 1 –is broadly representative of the majority of the selected journals.

*BMJ Open* has a total of 3,807 articles indexed, with output increasing each year since publication (2011 = 97, 2012 = 625, 2013 = 894, 2014 = 1,047, 2015 = 1,143). Starting with the first full year of publication (2012), these figures represent year on year increases of 43% (2013), 17% (2014) and 9% (2015).

Analysis of author nationality and affiliation highlights a number of key characteristics. There are 118 different author nationalities represented in the journal, with 34.0% of articles having at least one author from the UK. The USA and Australia are the next most common author nationalities, with 17.0% and 12.5% of articles respectively. No other nationality is responsible for authorship of more than 10% of articles, and analysis of author nationality by year reveals little variation over time. The most common author affiliations generally reflect the nationality findings, with the four most common (based on the proportion of articles with at least one author from that institution) all being to UK institutions (UCL = 4.2%, University of Oxford = 4.2%, King's College London = 3.0%, London School of Hygiene & Tropical Medicine = 2.8%). The University of Sydney (2.6%) is the fifth most common affiliation, with one Canadian (University of Toronto), one Swedish (Karolinska Institutet) and three further UK institutions (University of Bristol, University of Birmingham, and Imperial College London) completing the top 10. A further four UK and four Australian institutions rank between 11 and 20, but it is striking that despite being the second most common author nationality, the first USA institution (Harvard School of Public Health) ranks only 31^st^. This is perhaps a consequence of the much greater number of US institutions, so that submissions are spread across a broader number of research centres.

*BMJ Open* accepts submissions in a range of medical fields, including (but not limited to) “clinical medicine, public health and epidemiology … health services research, health economics, surgery, qualitative research, research methods, medical education, [and] medical publishing” (BMJ, 2016). *Scopus* does not allow for easy analysis of article subject by sub-discipline as it indexes all articles published in the journal under the “Medicine” subject area (one of 27 such broad subject areas), and the *BMJ Open* platform, unlike some other mega-journals such as *PLOS ONE* and *PeerJ*, does not offer sub-disciplinary classification.

As of mid-January 2016, the point at which all citation analyses were conducted, *BMJ Open* articles had been cited a total of 16,111 times. However in order to make meaningful comparisons between the citation data for all the mega-journals under review, citation analyses were conducted only for articles published in 2013, that year being the first for which all journals under review (with the exception of *Medicine*) had articles indexed by *Scopus*. The 894 articles published in *BMJ Open* in 2013 have been cited 5,867 times, giving a mean number of 6.6 citations per article. Following Björk & Catani [1], we also calculated the proportion of articles with 0, 1 and 2 citations, these figures being 10.0%, 12.5% and 13.6% respectively. The total proportion of articles with two or fewer citations is therefore 36%. In order to make sense of these data, similar calculations were made for three other comparable medical journals. There were three principal criteria for selecting comparison journals–subject area (broad-scope medicine), output (they should be similar in size to *BMJ Open*), and journal impact (used as a proxy for quality, the comparison journals should include a top, medium and low level journal). Given the broad scope of mega-journals, and the range of disciplines covered, it was decided to use Source Normalized Impact per Paper (SNIP) rather than Journal Impact Factor (JIF) as a measure of impact. SNIP seeks to measure a given journal’s contextual citation impact, and offers a means of comparing journals across different fields [16,17]. In the case of *BMJ Open* (2014 SNIP = 1.043), the journals selected for comparison were *PLOS Medicine* (3.207), *BMC Public Health* (1.229) and *Experimental and Therapeutic Medicine* (0.535). Table 2 shows some general information about these journals’ output and citations, along with the proportion of articles with 0, 1 and 2 citations.

**Table 2:** *BMJ Open* citation data compared to selected other medical journals.

						% of articles with *n* citations			
	2014SNIP	2013 Articles	Citations	Citations per article	Weighted mean SNIP of citing journals	0	1	2	≤ 2
*PLOS Medicine*	3.207	224	4,548	20.3	1.823	6.7	6.3	7.6	20.5
*BMC Public Health*	1.229	1283	6,809	5.3	1.323	7.5	11.7	14.4	33.6
***BMJ Open***	**1.043**	**894**	**5,867**	**6.6**	**1.303**	**10.0**	**12.5**	**13.6**	**36.0**
*Experimental and Therapeutic Medicine*	0.535	663	2,243	3.4	0.757	17.6	18.3	15.8	51.7

**Fig 1:**
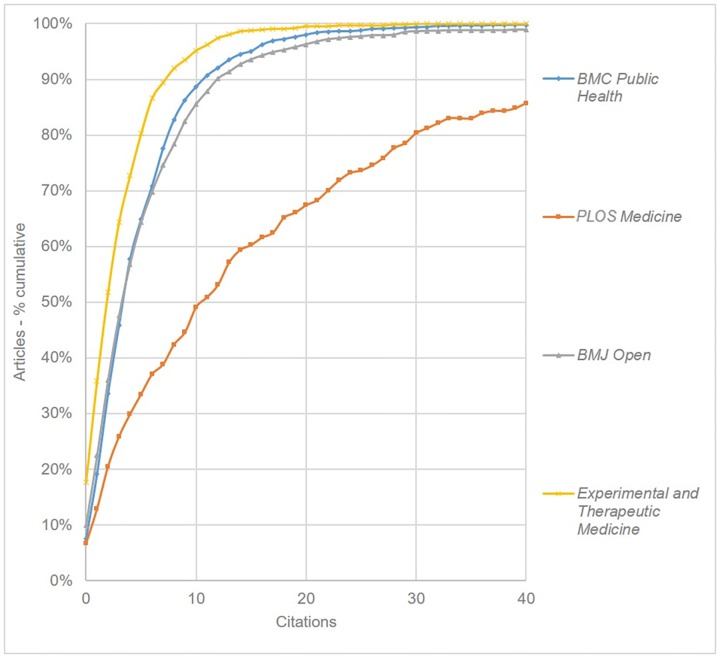
Cumulative citation frequency for *BMJ Open* and other selected medical journals (curves have been smoothed).

Taken in conjunction with Fig 1, which shows the cumulative citation frequency for each journal, we observe that *BMJ Open* presents a citation pattern very similar to that of *BMC Public Health*, a mid-ranking, relatively high-volume journal that operates with a traditional peer review policy. These two journals, together with *Experimental and Therapeutic Medicine*, are notably different from the longer established *PLOS Medicine* (which started publishing in 2004).

Consideration was also given to the quality of citing journals. This was estimated by calculating the weighted mean SNIP value for the top 50 citing journals. As shown in Table 2, in the case of *BMJ Open*, this was found to be 1.303, compared with 1.823 for *PLOS Medicine*, 1.323 for *BMC Public Health*, and 0.757 for *Experimental and Therapeutic Medicine*. Since the data failed the Levene test for equality of variances, a Welch analysis of variance (ANOVA) was employed, and showed significant variation (F(3, 3052) = 430.223, *p* < .001). The Games-Howell test was used for post hoc testing, as recommended by Ruxton *et al* [18], and showed that *BMJ Open* was significantly different to *PLOS Medicine* and *Experimental and Therapeutic Medicine* (both *p* < .001), but not significantly different to *BMC Public Health (p* = .896).

## Results and Discussion

### Mega-Journal Output

The total output to the end of 2015 of all eleven mega-journals is 178,075 articles, of which 44,820 were published in 2015. This represents an increase of 15% on the 2014 figure (38,995). *Scopus* has indexed a total of 1,826,143 articles published in 2015, meaning that the combined output of the mega-journals under review accounts for 2.5% of all articles.

**Fig 2:**
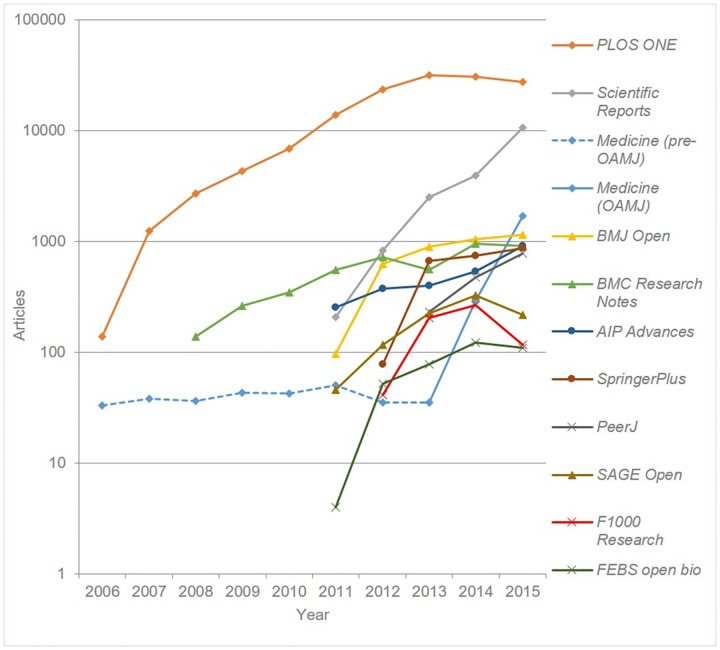
Mega-journal output by year (base 10 logarithmic scale used for y-axis).

Fig 2 plots the annual output of articles for each of the eleven OAMJs using a logarithmic scale. It should be noted here that since the journals were launched at different times of the year, output data for the first year of publication does not represent a full 12 month period, and is therefore best excluded from any analysis of growth. An initial review of growth suggests that for a number of journals an initial period of relatively fast growth is followed by a levelling off or slight decline in publishing output.

Table 3 presents the articles published in each OAMJ in 2015, and the percentage change since 2014. *PLOS ONE*’s output, which since 2013 has been around 30,000 articles per year, clearly dwarfs all other titles, with the recent exception of Nature’s *Scientific Reports*, which published 10,600 articles in 2015. While *PLOS ONE*’s output has declined by around 4,000 articles since its high of 31,404 in 2013, the concurrent growth of other mega-journals–most notably *Scientific Reports* and *Medicine*—has ensured that total OAMJ output has grown each year since 2006.

**Table 3:** OAMJ Output in 2015, and % change from 2014.

Journal	2015 Output	% Change from 2014
*PLOS ONE*	27,488	-9.3
*Scientific Reports*	10,600	+169.4
*Medicine*	1,694	+486.2
*BMJ Open*	1,143	+9.1
*BMC Research Notes*	904	-4.9
*AIP Advances*	902	+68.6
*SpringerPlus*	870	+17.1
*PeerJ*	777	+63.9
*SAGE Open*	215	-33.4
*F1000 Research*	117	-56.1
*FEBS Open Bio*	110	-9.1
*TOTAL*	44,820	+14.9

Table 3 suggests it might be reasonable to separate the eleven OAMJs into three groups: 1) those publishing over 10,000 articles in 2015 (*PLOS ONE* and *Scientific Reports*); 2) those publishing between 777 and 1694 articles in 2015 per year (*Medicine*, *BMJ Open*, *BMC Research Notes*, *AIP Advances*, *SpringerPlus*, and *PeerJ*), and 3) those publishing 215 or fewer (*SAGE Open*, *F1000 Research*, and *FEBS Open Bio*). It is perhaps instructive to compare the output of OAMJs with other journals. We note that of the 21,862 journals indexed in *Scopus*, only 3 (*PLOS ONE*, *RSC Advances*, and *Scientific Reports*) published more than 10,000 articles in 2015, while 134 journals (0.6%) published more than 1,000 articles and 420 (1.9%) more than 500 articles. Only 174 (0.8%) published more than the smallest group-2 journal—*PeerJ*’s 777 articles. We also note that one of the group-3 journals, *SAGE Open*, while substantially smaller than most of the other OAMJs listed here, is nonetheless the 16^th^ largest journal assigned to the “Social Science” subject area in *Scopus*.

It is also instructive to consider the range of growth and contraction figures presented in

#### Table 3

To put these figures in context, the total number of articles in *Scopus* is 0.9% lower in 2015 than 2014, a function perhaps of delays between article publication and their indexing in *Scopus*. While overall OAMJ output is rising, five of the eleven titles produced fewer articles in 2015 than 2014, including each of the three smallest OAMJs. The most striking increase in output belongs to *Medicine*, and is a function of the journal’s transition midway through 2014 from a small selective journal to a mega-journal model. The 1,694 articles it published in 2015 were more than its total output for the previous 50 years, and the journal now ranks fifth largest (by output) of all journals in *Scopus* assigned to the “Medicine” subject area. The only other journal to more than double in size is *Scientific Reports*, and it is useful to note that this growth was achieved without impacting the output of Nature’s two more prestigious journals, *Nature* and *Nature Communications* (both of which show slight increases in *Scopus* indexed output over the same period), i.e., the OAMJ has greatly increased the article output of the Nature stable of journals.

#### Discussion

Several issues emerge from this analysis of mega-journal output. Björk’s criterion relating to OAMJ size says that a mega-journal should have a “big publishing volume (or aiming for it)”. This leads inevitably to the question of how large a journal needs to be before it can be considered “big” (or indeed “mega”). Two mega-journals–*PLOS ONE* and *Scientific Reports*—clearly dwarf all others, and it is difficult not to view their competitors as comparatively small. The question is further complicated by disciplinary differences in journal size. Humanities and social science (HSS) journals, for example, are typically smaller than titles in the STM disciplines, again affecting notions of “bigness”. Perhaps the key question here is whether “big publishing volume” should be interpreted within the current scholarly communications context (in which case all but three of the eleven mega-journals are undeniably “big”) or instead whether the mega-journal phenomenon is intended to herald some new paradigm of *PLOS ONE*-size titles, in which case the current size of group 2 and 3 titles may come to be seen as nowhere near large enough to signify such a paradigm. Whether any of these titles, or indeed others not included in this analysis (for example *Royal Society Open Science* or *Heliyon*), will grow to group-1 size is open to question. Certainly it is notable that overall OAMJ output rose significantly (15%) between 2014 and 2015, despite a fall of 9% in *PLOS ONE*’s output. This rise is however largely attributable to just two journals–*Scientific Reports* and *Medicine*–and it is important to note that these journals have by far the largest JIFs of any of the OAMJs (5.578 and 5.723 respectively). While a purely bibliometric analysis is insufficient to prove the fact, it does appear likely that the comination of high impact factor and “objective” peer review policies make these journals particularly attractive publication venues for researchers. For this reason it will be interesting to observe future publication volumes for *Medicine*, should its impact factor decline (as seems likely). It remains to be seen which if any of the other OAMJs, which have in general shown relatively slow growth in recent years, are able to grow to group-1 size.

### Author Characteristics

Our analysis of OAMJ author characteristics focused on nationality and institutional affiliation. The ten most common author nationalities, and the proportion of articles with at least one author from these countries, are presented in S2 Appendix. Similar tables relating to institutional affiliation are provided as S3 Appendix. In total, 212 different author institutional nationalities were observed. USA authors were found to be the largest contributors to six of the eleven journals, with Chinese authors making the largest national contribution to three, and the UK and Japan each taking the top place for one journal. The US and UK are the only countries to appear in every top-10, with Germany (appearing in the list for 10 of the 11 journals), Canada and Japan (9), and China, Australia and Italy (8) all appearing frequently. Overall the table broadly reflects the general geographic distribution of authors across all research output, with *Scopus* showing the USA (21.9%) and China (19.8%) as the most common contributors to the 1,781,806 articles indexed in 2015, with the UK (6.5%), Germany (6.0%) and Japan (4.6%) all being in the top-10.

One question of interest is the extent to which mega-journals, as an OA innovation, facilitate the publication of research from institutions outside North America and Europe. It is notable that Chinese authors make up a very high proportion of authors for several OAMJs, reflecting the relatively recent boom in that country’s scholarly output [19]. Table 4 presents data relating to Chinese authorship for articles published in 2015 for all 11 journals, along with combined data for the other three BRIC nations (Brazil, Russia, and India) and the so-called “Next Eleven” (N-11) nations (Bangladesh, Egypt, Indonesia, Iran, South Korea, Mexico, Nigeria, Pakistan, the Philippines, Turkey and Vietnam). The table also shows the proportion of authors from these countries for all articles published in journals assigned the same Subject Area as the relevant OAMJ in *Scopus*. The prevalence of articles authored by researchers from these countries varies enormously. *Medicine*, *Scientific Reports* and *AIP Advances* all have disproportionately large numbers of Chinese authors, while four journals (*PeerJ*, *SpringerPlus*, *BMC Research Notes* and *F1000 Research*) have less than half the number of BRIC authors of the typical journal in their fields. Only one journal—*AIP Advances*–has a higher proportion of Brazilian, Russian or Indian authors than average, and seven of the eleven OAMJs have a smaller proportion of N-11 authors. Overall then these figures suggest that mega-journals are not attracting high numbers of articles from authors in developing countries.

**Table 4:** Contributions from Chinese, BRIC and Next-Eleven authors.

	% of articles with at least one author from:					
	China		Brazil, Russia, or India		N-11 Countries	
	OAMJ articles	All articles in same subject area(s)	OAMJ articles	All articles in same subject area(s)	OAMJ articles	All articles in same subject area(s)
*Medicine*	42.2	12.7	1.9	6.9	13.0	9.5
*AIP Advances*	40.6	27.7	14.3	14.2	14.1	11.2
*Scientific Reports*	39.2	30.0	5.5	12.7	7.4	9.5
*FEBS Open Bio*	19.1	21.0	8.2	9.7	3.6	9.2
*PLOS ONE*	18.9	14.2	6.7	8.7	6.6	9.9
*BMJ Open*	7.5	12.7	2.5	6.9	4.5	9.5
*PeerJ*	7.2	14.0	4.0	8.7	4.9	9.9
*SpringerPlus*	6.1	30.0	10.7	12.7	11.0	10.7
*BMC Research Notes*	1.6	13.8	4.4	7.7	8.7	9.6
*F1000 Research*	1.5	14.0	8.3	9.1	4.5	9.8
*Sage Open*	1.0	4.4	4.5	6.1	11.2	5.7

A year-by-year analysis was conducted to identify potential shifts in author demographics over time. Most journals show a slight rise in Chinese authorship, in line with wider trends in scholarly communications. Two titles (*Medicine* and *Scientific Reports*) however showed very large recent rises in Chinese authorship. *Medicine* was found to have the highest overall proportion of Chinese authored papers, and the longitudinal analysis revealed a dramatic increase in Chinese and Taiwanese authors following the journal’s transition to the mega-journal model.

Table 5 compares the ten most common nationalities for authors of articles published in *Medicine* between 2011 and 2013, with those published in the journal between June 2014 and December 2015 (June 2014 being the start of the mega-journal period). The proportion of articles with at least one Chinese author jumps from below 1% (there being only one Chinese authored article in the period 2011–2013) to over 40% once the journal becomes an OAMJ, so that a journal that once predominantly published research from USA and European (particularly French) medical research institutions, is now primarily publishing the work of Chinese researchers. However, it is also instructive to examine the number of articles published by authors from those European and US institutions in the mega-journal era, and compare these with the 2011–13 numbers. Table 6 lists the ten most prolific institutions in 2011–13, and compares their output in those years with June 2014–15. Despite the dramatic drop in the proportion of articles originating from these institutions, the actual number of articles published has remained stable or increased in the (shorter) mega-journal period, suggesting that the influx of Chinese authored papers has heavily supplemented rather than replaced papers submitted by US and European authors.

**Table 5:** *Medicine’s* most frequent contributing author nationalities pre- (2011–2013) and post- (June 2014–2015) mega-journal transition.

	2011–13		June 2014–2015	
	Country	%	Country	%
1	United States	37.5	China	40.9
2	France	25.8	Taiwan	15.9
3	Spain	16.7	United States	10.5
4	Japan	7.5	South Korea	8.1
5	Canada	4.2	Japan	5.5
6	Taiwan	3.3	Italy	4.2
7	Switzerland	2.5	France	3.7
8	United Kingdom	2.5	Spain	3.3
9	India	1.7	United Kingdom	2.6
10	Italy	1.7	Germany	2.4

**Table 6:** *Medicine*’s most frequent contributing author institutions for 2011–13, and their June 2014-December 2015 publications.

		2011–13		June 2014–2015		
	Institution	Articles	% all articles	Articles	% all articles	Change
1	The Johns Hopkins School of Medicine	8	6.7	6	0.3	-2
2	Université Pierre et Marie Curie	8	6.7	8	0.4	0
3	Hospital Universitari de Bellvitge	8	6.7	7	0.4	-1
4	Université Paris Descartes	8	6.7	15	0.8	+7
5	Université Paris 7- Denis Diderot	7	5.8	13	0.7	+6
6	Inserm	6	5.0	25	1.3	+19
7	Hôpital Pitié-Salpêtrière	6	5.0	10	0.5	+4
8	Hôpital Henri Mondor	6	5.0	7	0.4	+1
9	Massachusetts General Hospital	6	5.0	6	0.3	0
10	Universitat de Barcelona	5	4.2	8	0.4	+3

**Fig 3:**
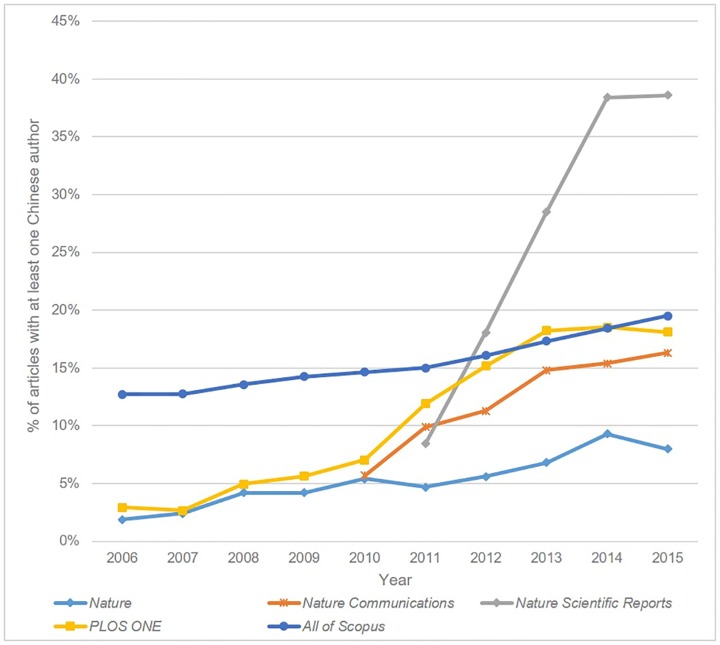
Proportion of articles with at least one Chinese author.

*Scientific Reports* was also observed to have a very high proportion of Chinese authored articles, with almost double the percentage of *PLOS ONE*. A comparison of the most common author affiliations for the two journals (see S3 Appendix) shows that the top 10 institutions contributing to *PLOS ONE* are mainly located in Europe and North America, while by contrast the list for *Scientific Reports* is dominated by Chinese and Japanese institutions. Fig 3 plots the yearly proportion of articles with at least one Chinese author for the three high-volume Nature titles (including *Scientific Reports*) and *PLOS ONE*. It is interesting to note that while all the journals show an increase in Chinese authorship over the last 10 years, reflecting the dramatic increase in Chinese research output, the increase for *Scientific Reports* is particularly steep. Chinese authors were responsible for just 18 articles in 2011, compared to 4,159 in 2015. We do however note that the proportion of Chinese authors remained relatively stable between 2014 (38.4%) and 2015 (38.6%), despite the large increase in article output over the same period (3,935 to 10,600)

Our analysis of author nationality also revealed that a relatively high proportion of *BMC Research Notes* articles have authors affiliated with African institutions. In total, 616 articles (13.9%) have at least one African author, with more than 300 different institutional affiliations observed for these articles. 185 of the 616 African-authored articles (30.0%) have at least one non-African co-author, with the USA (51) and UK (32) the most common collaborating nationalities. Table 7 shows a comparison of these data with those for another, similarly sized, medical mega-journal–*BMJ Open*. *BMC Research Notes* publishes a higher volume of African authored articles, from a greater range of African countries. It is particularly interesting to note the difference in the proportion of African authored articles that have a non-African co-author. These account for under one-third of African authored *BMC Research Notes* articles, but four-fifths of *BMJ Open*. This difference, and indeed the large number of African authored articles in general, can perhaps be at least partly explained by *BMC Research Notes’* current APC policy, which includes an automatic APC waiver for authors from “low or lower-middle income countries (according to World Bank criteria)” [20], whilst *BMJ Open* offers APC waivers only “in exceptional circumstances on request” [21].

**Table 7:** African authorship of articles in *BMJ Open* and *BMC Research Notes*.

	*BMC Research Notes*	*BMJ Open*
African countries represented	38	30
Articles with at least one African author	616	174
% of total journal article output that has African author	13.9	4.1
% of African authored articles with non-African co-author	30.0	80.0

#### Discussion

Overall, our analysis of OAMJ author nationalities and institutional affiliations reveals large variation across journals. Some OAMJs have clearly become popular for authors in specific geographic regions (*FEBS Open Bio*’s large Japanese author base, for example, or *BMC Research Notes*’ African contributors), while the distribution for other journals more closely matches that of scholarly output in general. Given the results for *Scientific Reports* and *Medicine*, it is tempting to attribute the dramatic increases in output for these journals in large part to the influx of Chinese authored articles. While in both cases Chinese is the most common author nationality, we note that the proportions are 38.6% and 40.9% respectively; i.e. a majority of authors are not Chinese. We also note that while *Scientific Reports*’ output more than doubled between 2014 and 2015, the proportion of Chinese authors remained constant. We might conclude therefore that while the factors driving increased submissions to mega-journals are applicable to all researchers, regardless of nationality, the particular sensitivity of Chinese authors to the JIF [22], provides additional incentive for their submission to certain OAMJs.

### Subject Areas

Although all eleven journals under discussion were deemed to meet Björk’s primary criterion of covering a “broad subject area”, there is clear variation in the disciplinary scope of the journals. Three journals (*PLOS ONE*, *Scientific Reports* and *SpringerPlus*) cover all of Science and Medicine, while others cover broad disciplines (e.g. *BMJ Open* for Medicine, *F1000 Research* for the life sciences). *FEBS Open Bio*, while still undeniably broad, is somewhat narrower than other OAMJs with its focus on “molecular and cellular life sciences”. The relative crudeness of *Scopus*’ subject area classification, which is applied at a journal level, precludes detailed analysis at a sub-disciplinary level, except for those OAMJ’s whose online platform offers such functionality. Here we note that two of the life science-orientated OAMJs, *PeerJ* and *F1000 Research*, appear to have evolved distinct specialisations within their broad categories. *PeerJ*’s platform, which it should be noted also includes content from its pre-print server, shows that almost a quarter (22.6%) of articles are classified as “Ecology”, while 19.4% are indexed as “Bioinformatics” and 15.6% as”Computational Biology”. In contrast, the *F1000 Research* platform (which includes articles that have not yet been reviewed) shows only 11.1% of articles under the broader field of “Plant Biology, Ecology & Environmental Sciences”, with the most commonly assigned subject being “Molecular, Cellular & Structural Biology” (31.4%).

*Sage Open* is the sole OAMJ of the eleven to accept only articles relating to the social sciences and humanities. The Sage platform allows for analysis by subject area, from which we determined that articles in the fields of education (16.8%), psychology (15.1%) and sociology (13.1%) were the most common. Articles assigned to the humanities subject area account for only 4.0% of total output (46 articles in total). It is interesting to observe here that *PLOS ONE* assigns 4,896 papers published in 2015 the “Social Sciences” subject category, an output that clearly dwarfs *Sage Open’s* social science publication numbers.

**Fig 4:**
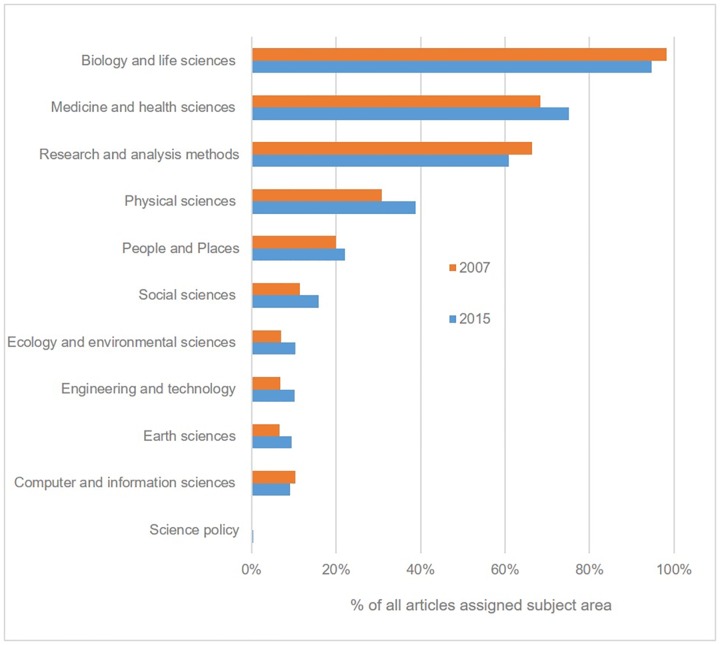
% of *PLOS ONE* articles indexed by top level subject areas.

Of particular interest are the three journals which claim to cover all scientific disciplines; *PLOS ONE*, *Scientific Reports* and *SpringerPlus*. In particular, we are interested in determining the extent to which any discipline or disciplines predominate. *PLOS ONE* states that it accepts submissions from “all disciplines within science and medicine” [23]. While *Scopus* does not classify articles by subject area, it is possible to use the *PLOS ONE* site itself to gather data about the subject areas assigned to published articles. PLOS operates a hierarchical, non-exclusive subject classification system with 11 high level subject areas [24]. Fig 4 shows the proportion of total articles assigned each of these subject areas in 2007 and 2015. It is immediately noticeable that a very high proportion of articles are assigned the “Biology and Life Sciences” subject area (94.6% in 2015). It is also interesting to observe the relatively high number of articles assigned the “Social Science” classification, despite the journal’s own criteria apparently limiting submissions to Science and Medicine. We also note that a high proportion of articles assigned the physical and social sciences subject areas must also be categorised as “Biology and Life Sciences”, suggesting that many submissions in these areas represent cross-disciplinary approaches to life science questions, rather than purely physical or social science questions. While we note a small decline in the proportion of life science articles between 2007 and 2015, and a corresponding slight increase for most other subject areas, the changes are not statistically significant. This perhaps suggests that the life sciences foci of PLOS’s initial journal offerings (*PLOS Biology* and *PLOS Medicine*) still influence the nature of submissions to the mega-journal.

The platform for *SpringerPlus* offers similar support for browsing content by subject area. “Biomedical and Life Sciences” (29.7%) and “Medicine” (37.4%) together account for around two-thirds of all output, with mathematics and physical science subject areas accounting for only 11.7% of all articles. Nature’s platform for *Scientific Reports* unfortunately does not offer comparable functionality for analysing subject areas. We can however use the *Scopus* subject areas for citing journals to reveal something about its disciplinary breadth. Table 8 shows the proportion of journals citing each of these three OAMJs that have been assigned to each of the 27 *Scopus* subject areas. It is important to note here that disciplinary variations in both citation rates [25] and *Scopus* coverage [15] are likely to skew these results. They are therefore presented not as estimates of subject coverage for each journal, but rather as a tool for comparing coverage, since the factors affecting the overall accuracy of the figures are constant for all three journals. The results suggest that *Scientific Reports* publishes a far greater proportion of articles in the physical sciences than either of the other two journals, both of which predominantly publish life sciences and medicine. While clearly there is some overlap between *PLOS ONE* and *Scientific Reports*, these results suggest that the two journals attract a high proportion of submissions from distinct disciplinary pools despite their declared coverage of a wide range of disciplines (see Table 1). It is also interesting to note that Springer’s stated rationale for closing *SpringerPlus* was that it covered “too wide a range of disciplines”, and that a “a one-size-fits-all journal is not the solution” [26]. Our results suggest that while the journal was broader than *Scientific Reports*, it covers a similar range of subjects to *PLOS ONE*.

**Table 8:** *Scopus* Subject Areas for citing journals as % of total citations for each journal (ranked by difference between *PLOS ONE* and *Scientific Reports*).

*Scopus* Subject Area of Citing Journal	*PLOS ONE (%)*	*Scientific Reports (%)*	*SpringerPlus (%)*
Medicine	57.5	20.6	51.5
Materials Science	1.5	28.3	2.8
Biochemistry, Genetics and Molecular Biology	47.5	22.2	30.7
Physics and Astronomy	1.6	25.7	2.6
Chemistry	3.7	23.6	4.7
Agricultural and Biological Sciences	22.1	8.3	19.0
Engineering	2.5	16.0	5.6
Immunology and Microbiology	13.5	4.4	9.6
Chemical Engineering	2.1	10.9	3.6
Multidisciplinary	2.0	9.1	5.0
Neuroscience	9.3	3.3	3.0
Energy	0.2	5.6	1.0
Pharmacology, Toxicology and Pharmaceutics	7.2	3.3	7.2
Mathematics	1.6	4.0	2.6
Psychology	2.6	0.8	1.6
Earth and Planetary Sciences	1.1	2.7	1.9
Nursing	1.3	0.0	2.6
Veterinary	1.2	0.0	1.6
Computer Science	2.4	3.6	4.4
Social Sciences	1.6	0.9	2.8
Arts and Humanities	1.2	0.5	0.4
Health Professions	0.7	0.0	2.0
Dentistry	0.4	0.0	0.4
Environmental Science	4.8	4.5	8.1
Business, Management and Accounting	0.1	0.0	0.2
Decision Sciences	0.1	0.0	0.4
Economics, Econometrics and Finance	0.1	0.0	0.3

#### Discussion

OAMJs exhibit significant variations not only in the intended scope of the journals, which range from broad sub-disiplines (*FEBS Open Bio*’s “molecular and cellular life sciences”) to overarching subject categories (e.g. the whole of Science and Medicine), but also in the extent to which certain sub-disciplines predominate. We present some evidence that journals with broadly similar stated scopes, in practice, have widely varying numbers of articles from particular sub-disciplines. It is beyond the scope of this paper to show precisely why this is the case, but we do offer some possible explanations. It seems natural that mega-journals launched by publishers with strong reputations in particular sub-fields might subsequently publish large numbers of articles in those areas, since authors in those disiplines are likely to have greater awareness of the journal (perhaps through targetted marketing). Familiarity with the publisher must also aid researchers in their evaluation of the OAMJ’s perceived quality or reputation. *PLOS ONE* is perhaps the clearest example of this phenomonon, PLOS’s first titles being *PLOS Medicince* and *PLOS Biology*, the two subject areas which have come to dominate the output of *PLOS ONE*. Less clear, and worthy of future study, is why particular specialisms have emerged in mega-journals without an obvious link between publisher and discipline. *SpringerPlus*, for example, has a disproportionately large life sciences and medicine output, despite publishing numerous high quality journals in the physical sciences. Another factor here may be the willingness or need for different academic communities to engage with Open Access publishing models in general, and mega-journals in particular. The evolution of the pre-print culture in some disciplines, particularly physics [27], may mean authors see less value in the OAMJ model, which promotes speed of publication and “objective” peer review as reasons to submit. The value of mega-journals in providing both is considerably diminished for a community accepting of the deposit of pre-print publications.

### Citation Analysis

Given the varying ages of the eleven OAMJs under review, the analysis of citation data was based primarily on citations for articles published in a single year—2013. This is the first year for which ten of the OAMJs were found to have articles indexed on *Scopus* (the exception being *Medicine*, which did not become an OAMJ until 2014). Since citations naturally increase over time, and there has been a maximum of only three years for these articles to accrue citations, the results naturally show lower citation rates than other studies which cover longer time periods. Table 9 presents the citation data for each journal, sorted by SNIP Value. It is interesting to note that *Scientific Reports* has a substantially higher SNIP value than the other OAMJs, while *F1000 Research* and *SAGE Open* have extremely low values. To put these figures in context, for the 20,202 journals assigned a 2014 SNIP value in *Scopus*, the 25^th^ percentile is a value of 1.067, and the median value is 0.680. Journals with a SNIP below 0.344 are in the bottom quartile. Only one OAMJ is in the top quartile, five are in the second quartile, above the median, two are in the third quartile and two in the bottom quartile. As might be expected, the crude measure of citations per article broadly reflects journal SNIP values, as does the proportion of articles with two or fewer citations. We find that for five of the OAMJs (*AIP Advances*, *BMC Research Notes*, *SpringerPlus*, *F1000 Research*, and *SAGE Open*) over half of all articles published in 2013 have received fewer than three citations, with *F1000 Research* and *Sage Open* having particularly high proportions of un-cited articles (42% and 60% respectively). It is also striking that *FEBS Open Bio*, despite having a relatively low mean number of citations per article, also has a small number of articles with zero citations.

**Table 9:** Citation overview of each OAMJ for articles published in 2013.

					% of articles with *n* citations			
OAMJ	SNIP (2014)	2013 Articles	Citations	Citations per article	0	1	2	≤ 2
*Scientific Reports*	1.402	2494	30,858	12.4	6.5	5.1	6.2	17.8
*BMJ Open*	1.043	894	5,847	6.5	6.0	11.0	11.9	28.9
*PLOS ONE*	1.034	31,404	223,541	7.1	6.2	8.4	10.0	24.5
*PeerJ*	0.818	229	1,260	5.5	12.2	9.6	14.0	35.8
*AIP Advances*	0.696	395	1,283	3.2	20.7	18.2	17.2	56.1
*BMC Research Notes*	0.683	555	1,569	2.8	23.4	22.1	15.6	61.2
*FEBS Open Bio*	0.516	78	295	3.8	9.0	14.1	25.6	48.7
*SpringerPlus*	0.511	666	1,567	2.4	32.2	20.8	15.6	68.6
*F1000 Research*	0.189	203	386	1.9	41.5	20.3	12.1	73.9
*SAGE Open*	0.174	222	156	0.7	60.1	23.7	9.2	93.0

**Fig 5:**
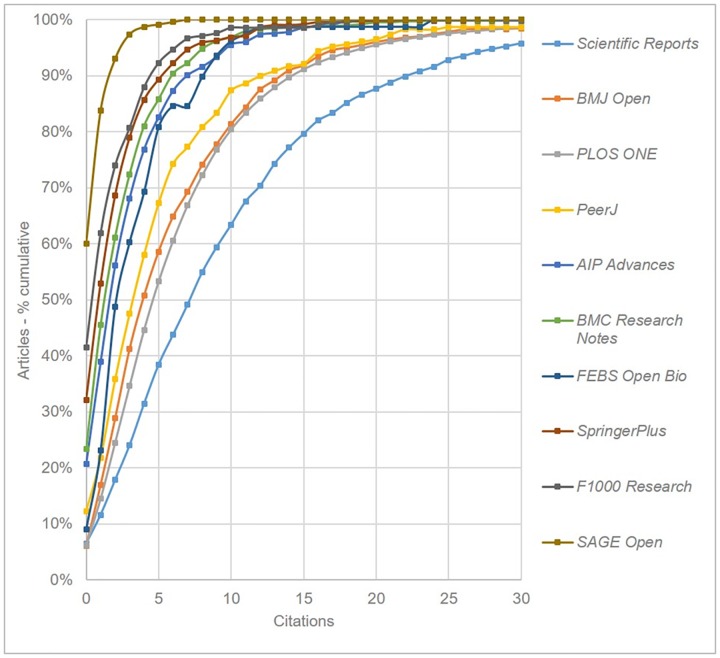
Cumulative citation distributions for all OAMJs based on articles published in 2013 (curves have been smoothed).

Following Björk & Catani [1] we plotted cumulative distribution function curves for each journal. These plots enable us to compare how evenly distributed citations are across the published articles. Fig 5 shows the data for each OAMJ, with *Scientific Reports* clearly seen to have a greater number of more highly cited articles than other titles. The graph suggests that *BMJ Open*, *PLOS ONE* and *PeerJ* share reasonably similar citation patterns, while *SAGE Open* is a clear outlier at the lower end of the scale, with no article having more than 6 citations. It is important however to view the *Sage Open* results in the context of the generally much lower citation rates in humanities and social science disciplines [28], as well as the proportionally poorer coverage of journals in these disciplines in *Scopus*.

**Fig 6:**
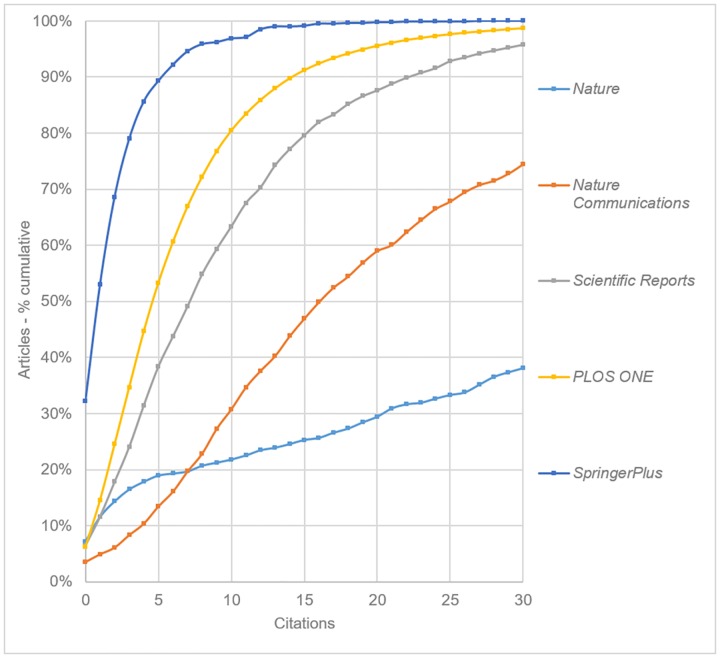
Cumulative citation distributions for the three general science OAMJs, plus selected comparison journals based on articles published in 2013 (curves have been smoothed).

Further analysis was conducted to contextualise these results, first by plotting the data for the three general science OAMJs (*PLOS ONE*, *Scientific Reports* and *SpringerPlus*) alongside two other, high-profile, conventional journal titles–*Nature* and *Nature Communications* (Fig 6).

We note first that *Nature* appears to have a surprisingly high number of articles with zero citations (7.2%)–higher in fact than the less prestigious *Nature Communications* and *Scientific Reports*. While *Scopus* filters were used to limit the data set only to articles, a manual analysis of the uncited *Nature* articles suggests that this filtering is not always successful. In particular, a number of comment and opinion pieces appear to be included. Nonetheless, 61.8% of *Nature* articles have accrued at least 30 citations, compared with 25.5% for *Nature Communications*, 4.2% for *Scientific Reports*, and 1.4% for *PLOS ONE*. Overall we see a clear delineation between the three Nature titles, with increasing levels of selectivity bringing increasing numbers and depth of citations. *SpringerPlus* is clearly the worst performing journal of this group, with 89.3% of articles having 5 or fewer citations.

**Fig 7:**
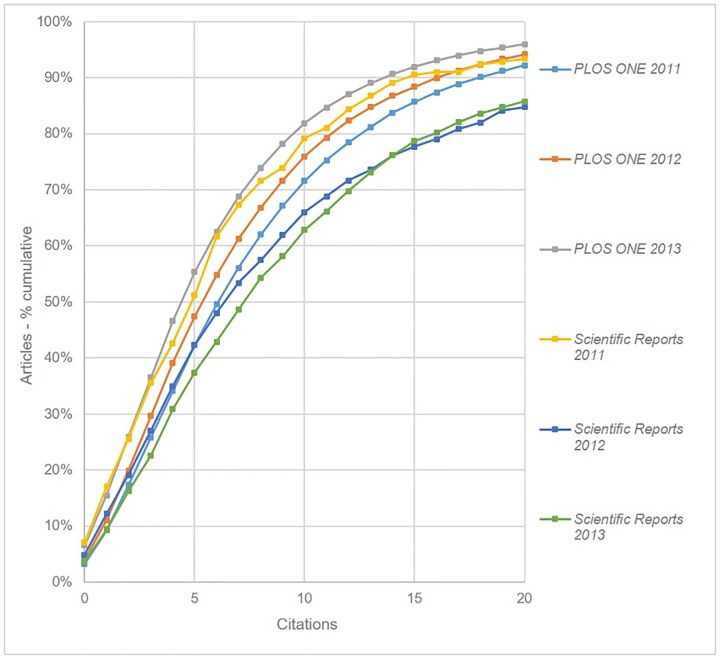
Cumulative citation distributions for *PLOS ONE* and *Scientific Reports* by year based on citations published before the end of the next calendar year (curves have been smoothed).

Comparing these results to those published by Björk and Catani [7] we observe that their plots showed the distribution of citations for *PLOS ONE* and *Scientific Reports* to be much closer to each other. They found, for example, that 25.5% of *Scientific Reports* articles, and 26.2% of *PLOS ONE* articles had two or fewer citations. Since their citation data were based on articles published between 2011 and 2013, rather than just in the latter year, additional analysis was carried out for these two journals for each year between 2011 and 2013. In order to make meaningful comparisons, for each year of publication only citations made before the end of the next-but-one calendar year were included. Thus for articles published in 2011, only citations in articles published prior to the end of December 2013 were considered. Fig 7 shows these plots, and reveals 2013 to be the year *Scientific Reports* performs best, and *PLOS ONE* worst. The graph also shows the extent to which *Scientific Reports*’ citation performance has improved since its 2011 launch, while *PLOS ONE*’*s* has declined over the same period. It will be interesting to see whether *Scientific Reports* maintains this trend for articles published in 2014, given the dramatic rise in publishing output that year.

**Fig 8:**
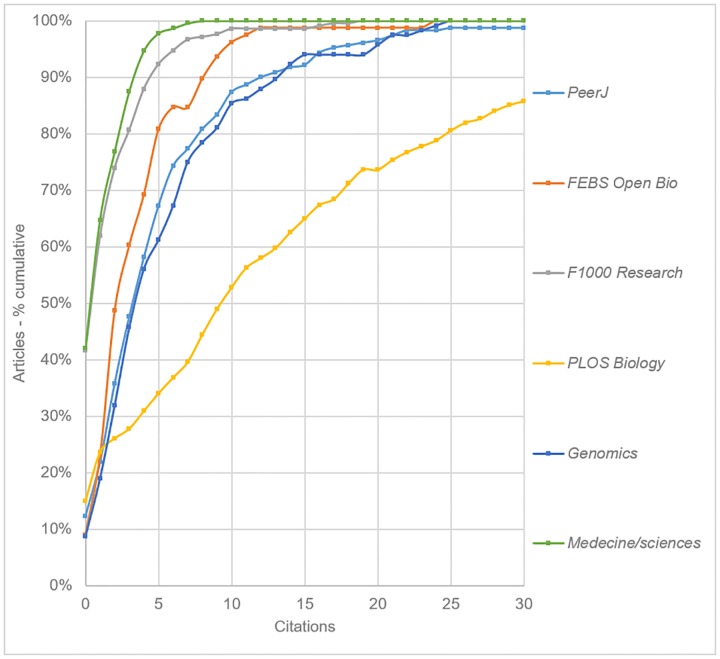
Cumulative citation distributions for the three life science OAMJs, plus selected comparison journals based on articles published in 2013 (curves have been smoothed).

Two further comparative analyses were conducted. Fig 8 shows the citation distribution for the three OAMJs with a focus on Biology (*PeerJ*, *FEBS Open Bio* and *F1000 Research*). and three similarly focused journals: *PLOS Biology* (2014 SNIP = 2.085), *Genomics* (0.827) and *Medecine/Sciences* (0.179). While *PLOS Biology* clearly has a greater number of highly cited articles than any of the OAMJs, *PeerJ* is found to present a similar profile to that of *Genomics*, a mid-ranking journal with traditional peer review policies. *F1000 Research* has a similar citation profile to the journal *Medecine/Sciences*–a French language journal with a similarly low SNIP value.

**Fig 9:**
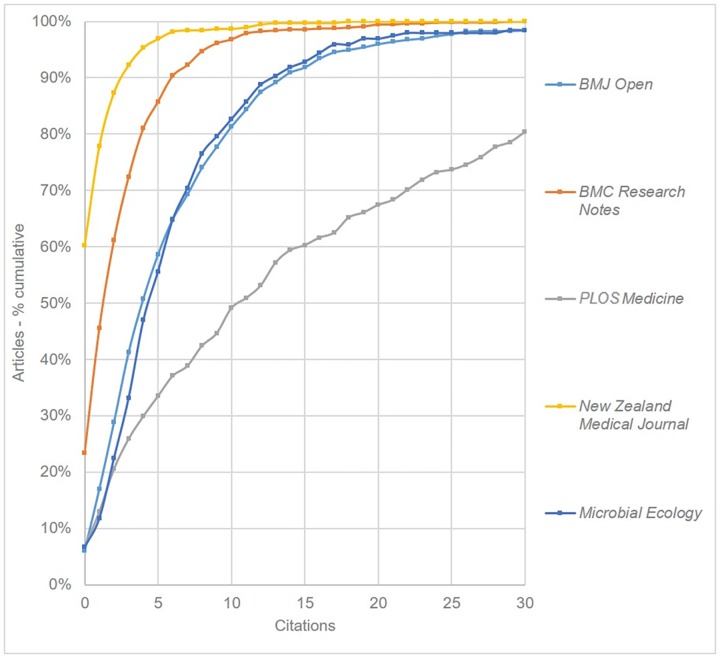
Cumulative Citation Distributions for two medical OAMJs, plus selected comparison journals based on articles published in 2013 (curves have been smoothed).

The final comparison was for the two medical OAMJs (*BMJ Open* and *BMC Research Notes*). Fig 9 also shows three comparison journals: *PLOS Medicine* (2014 SNIP = 3.207), *Microbial Ecology* (1.116) and *New Zealand Medical Journal* (0.273). We observe that while *PLOS Medicine* is much more highly cited than either of the OAMJs, *BMJ Open* has a similar citation pattern to *Microbial Ecology*, a traditional selective journal with a similar SNIP value. Both journals outperform the low-ranking but selective *New Zealand Medical Journal*.

Citation distributions were also calculated for *Medicine*, with a comparison made between articles published before and after the OAMJ transition. To ensure a fair comparison, we looked at citations to the 35 articles published in 2013 that occured before the end of 2014, and compared these to the citations to the 289 articles published in 2014 that occured before the end of 2015. For the 2013 articles, 20.0% were found to have zero citations, and 34.3% two or fewer. The post-megajournal articles were much less cited, with 46.4% having zero citations, and 82.7% two or fewer.

A further method of measuring the impact of research published in OAMJs is to analyse not just the number of citations, but the impact or quality of the citing journals themselves. Table 10 presents the Weighted Mean SNIPs for the top 50 citing journals for each of the ten OAMJs, based on an analysis of citations for all articles published between 2011 and 2014 (*Medicine* is excluded because it was not an OAMJ for most of this period). The table also includes data relating to the proportion of citing journals that are Open Access (OA), and the proportion of citations that originate from OA journals. We find that the journals citing articles published in *Scientific Reports* have the highest mean SNIP, and six OAMJs have higher values than *PLOS ONE*. While *F1000 Research* has a low SNIP value—due to its large proportion of infrequently cited articles—the weighted mean SNIP of its citing journals is relatively high. A Welch ANOVA showed significant variation (F(9, 1612) = 1029, *p* < .001) across all journals, and a post hoc Games-Howell test showed that *Scientific Reports* was significantly different to all other OAMJs (all to *p* < .001), while *PLOS ONE* was not significantly different to *F1000 Research*, *BMC Research Notes*, *FEBS Open Bio or Sage Open* (all *p* >.23). Considerable variation is observed in the proportion of citing journals and articles that are OA, which may in large part be explained by different disciplinary attitudes towards Open Access. We note for example that the journals with the lowest proportion of OA citing journals–*SAGE Open*, *Scientific Reports* and *AIP Advances*–primarily cover disciplines (the social and physical sciences) that have among the lowest rates of Gold- OA journal publishing [29].

**Table 10:** Weighted Mean SNIP of top 50 citing journals, and proportion of OA citing journals and citations. Based on citations for articles published between 2011 and 2014.

OAMJ	SNIP (2014)	Weighted Mean SNIP of citing journals	% Citing journals that are OA	% Citations in OA Journals
*Scientific Reports*	1.402	1.616	20.0	34.8
*BMJ Open*	1.043	1.303	44.0	69.4
*AIP Advances*	0.696	1.270	14.0	18.9
*PeerJ*	0.818	1.268	46.0	67.9
*F1000 Research*	0.189	1.230	42.0	64.3
*BMC Research Notes*	0.683	1.143	54.0	74.6
*PLOS ONE*	1.034	1.160	46.0	71.4
*FEBS Open Bio*	0.516	1.092	32.0	47.8
*SAGE Open*	0.174	0.965	24.0	41.1
*SpringerPlus*	0.511	0.943	38.0	59.1

It is also interesting to compare figures for *Medicine* before and after its transition to an OAMJ. The weighted mean SNIP of citing journals for the final two years of the journal’s traditional existence was 1.428, significantly higher than the OAMJ figure of 0.974 (t(796) = 5.86, *p* < 0.01). Articles published in the OAMJ incarnation of *Medicine* therefore appear to be cited in lower impact journals than those published before the transition. We also note that the proportion of OA citing journals (20%), and proportion of citations in OA journals (22%) are both much lower than after the transition (38% and 52% respectively). It is thus possible that the transition may have adversely affected the overall impact of the journal but enhanced its recognition within the OA community.

#### Discussion

Our findings offer broad support for Björk and Catani’s [1] suggestion that OAMJs’ citation distributions are often not dissimilar to those of some traditional journals, despite their new approach to peer review. In particular, we observe that the distributions are similar to journals of similar impact in the same discipline, but with much higher numbers of infrequently cited articles than highly prestigious and selective journals. The two largest OAMJs also have the best citation distributions, with *Scientific Reports* articles being the most cited of any OAMJ.

Understanding why citation rates differ so widely between OAMJs is challenging. While disciplinary variation is clearly a factor, our analysis suggests that even journals in broadly similar subjects exhibit quite different proportions of infrequently cited articles. The perceived quality of the OAMJ for the citing author is perhaps one factor here, with authors potentially more likely to cite articles from *PLOS ONE* (the world’s largest journal and best known OAMJ) or *Scientific Reports* (published by Nature) than other less well-known mega-journals. It is also possible that OAMJs with more efficient processes for capturing submisssions rejected by other more selective journals within the same publisher stable perform better, the argument being that authors might opt for a path of least resistance rather than prolong the publication process by re-submittting to selective journals from other publishers. While not offering a true cascading review system, Nature does facilitate the resubmission of articles rejected from Nature titles to *Nature Communications* and *Scientific Reports* [30], and it seems likely that further research into the number of such re-submitted articles, and their citation performance, could help explain the latter journal’s relatively high citation rates.

At the heart of this discussion, however, is the fact that all eleven OAMJs claim to operate review policies that evaluate articles only on the basis of their scientific soundess. It follows, therefore, that the better performing mega-journals are either somehow attracting more citable submissions, or that the scientific soundness peer review policy is being implemented differently across different OAMJs. Better understanding exactly how the “objective” peer review process is operated–how reviewer guidelines explain the policy to reviewers, how reviewers interpret those guidelines, and how their reviews are interpreted by editors–may shed light on the extent to which these policies impact citation rates.

## Conclusions

In this paper we have presented the first broadly-based quantitative review of four key characteristics of mega-journals; the numbers of published articles, the national and institutional characteristics of the authors of those articles; the subject areas covered by the journals; and the citations to them. We found that the total output of the eleven mega-journals under review grew by 14.9% between 2014 and 2015, with two OAMJs–*PLOS ONE* and *Scientific Reports*–together accounting for 85.0% of the 2015 total. This growth, achieved despite a drop in the number of *PLOS ONE* articles, is largely attributable to *Scientific Reports* and *Medicine*. In terms of describing the defining characteristics of OAMJs we suggest that determining what constitutes a “large” article output will depend on whether mega-journals are considered relative to the current journal publishing context of any given discipline, or instead whether they are considered a paradigm-shifting phenomenon. With regard to author characteristics, we noted substantial variation in the geographical distribution of authors, with some OAMJs clearly proving popular in specific regions. Several journals (particularly *Scientific Reports* and *Medicine*) have a relatively high proportion of Chinese authors, and we suggest this may be linked to these journals’ high JIFs. The mega-journals were also found to vary in subject scope, and we found that several journals publish disproportionately high numbers of articles relating to certain sub-disciplines. Most notably, *PLOS ONE*, despite accepting submissions from all areas of science, is predominantly publishing articles in the life sciences. *Scientific Reports*, in contrast, appears to have a more even spread of articles, including many relating to the physical sciences. Our citation analsysis offers support for Björk & Catani’s [1] suggestion that OAMJs citation distributions can be similar to those of traditional journals. We also note a clear distinction in citation distributions between *Scientific Reports*, proportionally the most highly cited mega-journal, and the two other broad scope Nature journals.

Open Access Mega-Journals, while a relatively recent phenomenon, have emerged as an important part of the scholarly communication landscape. It seems clear however that while the term is useful as a means of grouping journals which share a set of key characteristics, there is no such thing as a “typical” mega-journal. Our analysis shows substantial variations not only in terms of size, but also disciplinary area, author characteristics, and citation profiles. It is often the case with bibliometric studies that they raise as many questions as they answer, and we suggest that further research into some of the issues raised in this study would greatly aid a deeper understanding of the mega-journal phenomenon. For example we believe that investigation of publisher rationales for launching OAMJs, and their long-term strategic aims for the journals, would allow us better to contextualize some of the data presented in this paper. We also suggest that further work be done to investigate how soundness-only peer-review is understood by reviewers, and what impact if any the new approach has on their reviews. Finally, better understanding academics’ perceptions of OAMJs, and their incentives for publishing in them, would tell us more about their potential future.

## Supporting Information

S1 Appendix Results of review of potential OAMJs. (XLSX)

S2 Appendix Most common author national affiliations for each OAMJ. (XLSX)

S3 Appendix Most common author institutional affiliations for each OAMJ. (XLSX)
